# Surgical outcomes of drug-refractory infantile epileptic spasms syndrome and related prognostic factors: a retrospective study

**DOI:** 10.1186/s42494-024-00176-1

**Published:** 2024-11-01

**Authors:** Xueyan Cao, Xin Ding, Fengjun Zhu, Li Chen, Yan Chen, Yang Sun, Lin Li, Zeshi Tan, Cong Li, Yi Yao, Qiru Su, Dezhi Cao

**Affiliations:** 1 Surgical Division of Epilepsy CenterFutian DistrictGuangdong Province, Shenzhen Children’s Hospital, 7019 Yitian Road, Shenzhen City, 518038 China; 2 Neurology Department, Shenzhen Children’s Hospital, Shenzhen, 518000 Guangdong Province China; 3 Department of Clinical Research, Shenzhen Children’s Hospital, Shenzhen, 518038 Guangdong Province China

**Keywords:** Drug-refractory epilepsy, Hemispherotomy, Post-surgical outcome, Structural lesions, Surgical treatment, Infantile epileptic spasms syndrome

## Abstract

**Background:**

In this study, we aimed to assess the efficacy of surgical treatment in children with drug-refractory infantile epileptic spasms syndrome (IESS) and examine the factors influencing the post-surgical outcomes.

**Methods:**

The clinical data of 30 children (18 males and 12 females) with epileptic spasms (ES) who underwent surgery at the Epilepsy Center of Shenzhen Children’s Hospital between June 2018 and June 2020 were retrospectively analyzed. Post-surgical outcomes were evaluated using the Engel Epilepsy Surgery Outcome Scale. Scalp electroencephalography and developmental quotient were assessed preoperatively and postoperatively. Univariate analysis and exact logistic regression analyses were used to identify the factors affecting the postoperative efficacy.

**Results:**

Of the 30 patients who underwent surgical resection, 22 (73.3%) achieved Engel’s class I–II outcomes. Additionally, motor and cognitive functions improved in 14 patients (46.7%). The development of 12 (40%) patients remained at the preoperative development level. The median number of antiseizure medications taken preoperatively was 5.27 (range 2–10), which decreased to 1.90 (range 0–4) at the last follow-up. Seizure duration, etiology, positive positron emission tomography-magnetic resonance imaging (PET-MRI), surgery type, and lesion location were significantly correlated with the postoperative efficacy (*P* < 0.05). Positive PET/MRI findings and lesion location predicted independently the postoperative outcomes. Permanent impairments of motor or language function were rare, with only two cases reporting hydrocephalus and one reporting hemiplegia.

**Conclusions:**

Surgery is an effective treatment option for children with IESS. Early referral and comprehensive preoperative evaluation are essential for identification of surgically treatable structural lesions. The primary surgically treatable cause is cortical malformation, followed by perinatal brain injury. Hemispheric disconnection is a preferred surgical approach. Positive PET/MRI findings and lesion location predicted the postoperative outcomes.

**Supplementary Information:**

The online version contains supplementary material available at 10.1186/s42494-024-00176-1.

## Background

Infantile epileptic spasms syndrome (IESS) is an age-dependent epileptic encephalopathy that typically affects children between 3 months and 2 years of age. The incidence of IESS is estimated to be 2–5 per 10,000 liveborn infants, with a slightly higher prevalence among male individuals [1]. This condition is characterized by a triad of epileptic spasms (ES), hypsarrhythmia on internal scalp electroencephalography (EEG), and developmental stagnation or regression. ES are seizures classified into focal, generalized, and onset-unknown types according to the 2017 Classification by the International League Against Epilepsy (ILAE) [2]. ES with a focal onset of IESS often indicates the presence of focal epileptogenic foci. Additionally, infants with ES may experience focal seizures, especially in cases with structural etiology. These cases are often drug-refractory and should be promptly referred for surgical evaluation. Surgical intervention may lead to seizure remission and improvements in neurocognitive function [3–5]; however, there are few case reports regarding the efficacy of surgery and the factors influencing the prognosis of children with epileptic encephalopathy [6].

This study was set out to retrospectively investigate the efficacy of surgical treatment in children with drug-refractory IESS and examine factors that influence the post-surgical outcomes. Furthermore, we will discuss the indications, surgical approaches, necessity of secondary-stage surgery, and factors related to the efficacy and outcomes, to elucidate the timing, effectiveness, and safety of surgery in children with IESS.

## Methods

### Patients

Patients with IESS who underwent surgical treatment at Shenzhen Children's Hospital between June 2018 and June 2020 were retrospectively reviewed. This study was approved by the Ethics Committee of Shenzhen Children's Hospital (202004502). Written or verbal informed consent was obtained from parents of all patients. The inclusion criteria included: (1) diagnosis of IESS based on the ILAE Classification 2017; (2) meeting the diagnostic criteria for drug-refractory epilepsy [7]; (3) onset age between 1 month and 3 years; and (4) regular follow-up over 1 year after epilepsy surgery at Shenzhen Children’s Hospital. The exclusion criterion was inability to return for scheduled follow-up owing to family reasons.

### Preoperative evaluation

A comprehensive preoperative evaluation was carried out for all enrolled children with IESS, which consisted of two stages (Fig. S1). The first stage included localization of the epileptogenic zone (EZ) and identification of eloquent brain areas based on detailed medical history and using noninvasive methods such as EEG, cranial magnetic resonance imaging (MRI), cranial positive positron emission tomography (PET) scan, PET-MRI analysis, and neuropsychological assessments. If EZ could not be identified by noninvasive evaluations but was highly suspected of focal initiation, or if EZ might involve functional areas, then the second stage of evaluation was initiated. In this stage, patients were implanted with intracerebral electrodes for EEG recordings to determine the range of EZ and its relationship with eloquent brain areas. Palliative treatment was considered if the EZ was not localized, as surgical resection is difficult, carries high surgical risk, creates high traumatization, and may cause serious neurological deficits. In this study, stereoelectroencephalography radiofrequency thermocoagulation (SEEG-RF-TC) or corpus callostomy (CC) was chosen as palliative treatment or as a diagnostic treatment before surgical resection. Curative surgery was performed upon clear determination of the EZ and the lesion range, ensuring no overlap with critical functional areas. Hemispheric disconnection were chosen for those with structural lesions involving one entire hemisphere.

Resections, disconnections, or local thermocoagulation was performed using protocols established during the preoperative evaluation. When necessary, an electrocorticogram (ECoG) was used to monitor the abnormal discharge range. After surgery, intravenous levetiracetam was used to prevent transient seizures due to postoperative cerebral edema. Additionally, adjustments were made for preoperative oral anti-seizure medications (ASMs), and patients received symptomatic and supportive treatments including hemostasis and sedation after surgery.

### Follow-up and efficacy analysis

Follow-up was performed at 1, 3, 6, and 12 months postoperatively, followed by regular annual check-ups. Based on the Engel Epilepsy Surgery Outcome Scale, the patients were classified into the Engel I–II and III–IV groups. The Engel I–II group was categorized as the favorable outcome group, whereas the classes III–IV group was classified as the poor outcome group.

### Statistical analysis

All analyses were performed using the Statistical Product and Service Solutions 26 (IBM Corp., Armonk, NY) and the Statistical Analysis System (Cary, NC) softwares. Continuous variables exhibiting non-normal distribution are presented as the median with range (interquartile range). Categorical variables are shown as *n* (%). Data with normal distribution are presented as mean ± standard deviations. Measurement data with non-normal distributions were analyzed with non-parametric tests, and comparisons of count data were made using the Chi-square test or Fisher's exact probability method. Univariate analysis and exact logistic regression analyses were used to analyze factors affecting post-surgical efficacy. Odds ratios and 95% confidence intervals were also calculated. The level of significance was set at *P* < 0.05.

## Results

### Patient characteristics

A total of 30 patients (18 males and 12 females) underwent epilepsy surgery. The age of seizure onset was 4.0 (0.3–12.0) months, and the age at the time of surgery was 17.0 (2.6–45.0) months. The duration from seizure onset to surgical treatment was 14.0 (0.6–40.0) months. Among them, 7 cases received surgery within 6 months of age, 5 cases between 6 to 12 months, 15 between 12 to 36 months, and 3 after 36 months. The number of ASMs taken preoperatively ranged from 2 to 10, with a median of 5.27 that decreased to 1.90 (range 0–4) postoperatively.

The preoperative seizure types included epileptic spasms in 9 patients and a combination of epileptic spasms and focal seizures in 11 patients (36.7%). The other 10 patients experienced more than two seizure types. Preoperatively, 29 children experienced daily seizures, with 20 cases (69.0%) reporting three seizures or more per day, and 8 cases (27.6%) experiencing more than 10 seizures per day.

### Postoperative efficacy and influencing factors

The follow-up duration was 15–38 (average, 25) months. According to the Engel Epilepsy Surgery Outcome Scale, 22 cases had favorable outcomes, including Engel I outcome was achieved in 16 cases (72.7%) and Engel II in 6 cases (27.2%). Seizure duration, etiology, positive PET/MRI findings, surgical type, and lesion location were factors significantly affecting the post-surgical efficacy (*P* < 0.05) **(**Table 1**)**.

**Table 1:** Comparison of relevant clinical indicators affecting the surgical efficacy in children [*n* (%)]

Influencing factor	Effective group (*n* = 22)	Less effective group (*n* = 8)	*χ*^*2*^	*P*-value	OR (95%CI)
Sex			0.455	0.396	
Male	14 (63.6)	4 (50.0)			1.00
Female	8 (36.4)	4 (50.0)			1.75 (0.34–8.98)
Age at seizure onset			0.341	0.452	
< 6 months	14 (63.6)	6 (75.0)			1.00
6 months–1 year	8 (36.4)	2 (25.0)			0.58 (0.09–3.60)
Age at surgery			2.744	0.108	
< 1 year	10 (45.5)	1 (12.5)			1.00
> 1 year	12 (54.5)	7 (87.5)			5.83 (0.61–55.74)
Seizure duration			4.224	0.047	
Within 1 year	12 (54.5)	1 (12.5)			1.00
Over 1 year	10 (45.5)	7 (87.5)			8.40 (0.88–80.27)
Etiology			5.408	0.020^*^	
Innate structure	17 (77.3)	4 (50.0)			
Acquired structure	5 (22.7)	1 (12.5)			-
Heredity	0 (0.0)	3 (37.5)			
Seizure type			2.078	0.161	
Epileptic spasm	5 (22.7)	4 (50.0)			1.00
Combined with other types of seizure	17 (77.3)	4 (50.0)			0.29 (0.05–1.62)
MRI			0.545	0.405	
Positive	19 (86.4)	6 (75.0)			1.00
Negative	3 (13.6)	2 (25.0)			2.11 (0.28–15.77)
PET-MRI			5.514	0.048	
Positive	21 (95.5)	5 (62.5)			1.00
Negative	1 (4.5)	3 (37.5)			12.6 (1.07–148.13)
Surgery type			3.996	0.046^*^	
Hemispherectomy	10 (45.5)	3 (37.5)			-
One lobe/multilobar resection	12 (54.5)	1 (12.5)			
Palliative operation	0 (0.0)	4 (50.0)			
Lesion location			12.692	0.003	
Focal/unilaterality	22 (84.6)	4 (15.4)			1.00
Bilateral	0 (0.0)	4 (15.4)			0.15 (0.06–0.38)

^*^Linear trend testing, palliative surgery (including stereotactic radiofrequency thermocoagulation or corpus callosotomy)

*CI* confidence interval, *MRI* magnetic resonance imaging, *OR* odds ratio, *PET* positron emission tomograph

Furthermore, multifactorial logistic regression analysis was performed on factors associated with statistical significance at the test level of α = 0.05 for one-way analysis (Table 1). The maximum likelihood method of estimation suggested that there was complete separation of the data, and the system warned that conventional logistic regression was invalid. Therefore, the prognosis of 30 children operated on for IESS was analyzed by exact logistic regression using the Statistical Analysis System software. The results of the joint test showed statistically significant associations of PET-MRI and lesion location with postoperative outcomes (*P* < 0.05) (Tables 2–3). Receiver operating characteristic (ROC) curves were constructed to predict the effects of positive PET-MRI findings and lesion location on the surgical efficacy. The area under the ROC curve for the prediction model was 0.9261, indicating high accuracy of our predictive model (Fig. S2).

**Table 2:** Results of the conditional exact test (*α* = 0.05)

Factor	Test	Statistics	Exact *P-*value	Mild *P-*value
Joint	Conditional score	20.5741	< 0.0001	< 0.0001
	Conditional probability	0.000015	< 0.0001	< 0.0001
PET-MRI	Conditional score	12.4096	0.0060	0.0030
	Conditional probability	0.00589	0.0060	0.0030
Lesion location	Conditional score	19.0909	0.003	0.0002
	Conditional probability	0.000334	0.003	0.0002

Mild *P-*value: *P-*value adjusted for discrete distribution. *MRI* magnetic resonance imaging, *PET* positron emission tomography

**Table 3:** Exact parameter and OR estimates (*α* = 0.05)

Factor	Point estimation	*OR* value	95%CI	*P-*value
PET-MRI	− 3.7186	0.024	(< 0.001–0.527)	0.0119
Lesion location	− 1.9639*	0.140*	(0.000–0.378)	0.0003

^*^median-unbiased estimator

*CI* confidence interval, *MRI* magnetic resonance imaging, *PET* positron emission tomography

Postoperative complications occurred in three patients. The complications included hydrocephalus in two patients and hemiplegia in one patient after stereotactic radiofrequency thermocoagulation. In conjunction with the neurodevelopmental scale test, gross motor and fine motor skills were improved in 14 children (46.7%). Meanwhile, the development of 12 children (40%) remained at the preoperative developmental level.

### Etiology and pathological analysis

The etiology of this group of cases was classified as follows according to the ILAE Classification: (1) genetic etiology, including clear causative *STAMBP* compound heterozygous gene mutation in one case, and suspected causative gene mutations in two cases, of whom the clinical significance of the mutations was unclear, but clinical history strongly suggested a genetic etiology; and (2) structural etiology, including congenital structural etiology in 21 cases (accounting for 70%), comprising 16 cases of focal cortical dysplasia, one case of cerebral tumor, and four cases of developmental malformation of the brain; and acquired structural causes including perinatal brain injury in five cases, sequelae of hypoxic-ischemic encephalopathy in three cases, sequelae of intracranial hemorrhage in two cases, and necrotizing encephalopathy sequelae in one case.

Pathological examination revealed focal cortical dysplasia (FCD) alone in 17 patients (including 12 cases of FCD Ia, one case of FCD Ib, one case of FCD IIa, one case of FCD IIb, one case of FCD IIIa, and one case of FCD IIId), glial scarring in 7 patients (encephalomalacia combined with glial scarring in one case), and hemimegalencephaly and ganglioglioma in one case each. A total of 26 patients underwent resection or disconnective surgery. Based on the post-surgical pathological findings, the cases were divided into the FCD and the non-FCD groups. A comparison of the pertinent clinical characteristics between the two groups revealed that the disease duration in the FCD group was longer than that in the non-FCD group. This suggested that the patients in the FCD group had more atypical clinical features, a more insidious etiology, and a longer time from symptom onset to receiving surgical intervention (Table 4). In addition, the various pathological subtypes of FCD did not significantly impact the postoperative outcomes (Table 5).

**Table 4:** Comparison of clinical characteristics of patients with different pathological types [*n* (%)]

Influencing factor	FCD group (*n* = 17)	Non-FCD group (*n* = 9)	*P-*value
Sex			0.189
Male	12 (70.6)	4 (44.4)	
Female	5 (29.4)	5 (55.6)	
Age at seizure onset			0.365
< 6 months	12 (70.6)	5 (55.6)	
6 months–1 year	5 (29.4)	4 (44.4)	
Age at surgery			0.079
< 1 year	5 (29.4)	6 (66.7)	
> 1 year	12 (70.6)	3 (33.3)	
Seizure duration			***0.048***
Within 1 year	6 (35.3)	7 (77.8)	
Over 1 year	11 (64.7)	2 (22.2)	
Seizure type			0.413
Epileptic spasm	6 (35.3)	2 (22.2)	
Combined with other types of seizure	11 (64.7)	7 (77.8)	
MRI			0.159
Positive	13 (76.5)	9 (100.0)	
Negative	4 (23.5)	0 (0.0)	
PET-MRI			0.159
Positive	13 (76.5)	9 (100.0)	
Negative	4 (23.5)	0 (0.0)	
Surgery type			0.500
Hemispherectomy	9 (52.9)	4 (44.4)	
Non-hemispherectomy	8 (47.1)	5 (55.6)	
Outcome			0.420
Engel (I–II)	13 (76.5)	8 (88.9)	
Engel (III–IV)	4 (23.5)	1 (11.1)	

*MRI* magnetic resonance imaging, *PET* positron emission tomography

**Table 5:** Effects of pathological type of FCD on postoperative efficacy [*n* (%)]

Influencing factor	Effective group (*n* = 13)	Ineffective group (*n* = 4)	*P-*value
Histopathology			0.70
FCD type 1	10 (76.9)	3 (75.0)	
FCD type 2	1 (7.7)	1 (25.0)	
FCD type 3	2 (15.4)	0 (0.0)	

*FCD* focal cortical dysplasia

## Discussion

IESS is a prevalent developmental epileptic encephalopathy that manifests during infancy and childhood. Children with IESS often experience drug-refractory seizures. Currently, the preferred treatment methods for IESS include adrenocorticotropic hormones and vigabatrin, followed by a variety of broad-spectrum ASMs. However, it is noteworthy that despite these treatments, approximately 66.7% of patients with IESS have uncontrolled seizures and psychomotor retardation [8]. The prognosis of patients with IESS, especially those with structural etiologies, can be greatly improved if the EZ can be identified and surgically resected at an earlier stage. In a randomized trial of pediatric epilepsy surgery in patients with IESS, a significantly higher proportion of patients achieved seizure freedom at 1 year postoperatively in the surgery group with drug-resistant epilepsy receiveing brain surgery along with appropriate medical therapy, compared to those with medical therapy alone (77% vs. 7%, *n* = 116) [9]. Here, we analyzed postoperative outcomes in 30 children with IESS and drug-refractory epilepsy. Our findings revealed that seizures were effectively controlled in 22 patients (73.3%) with IESS after surgery. The finding was consistent with a previous study reporting seizure-free rates of 50–70% after infantile epilepsy surgery [10]. Furthermore, four patients discontinued ASMs after surgery and remained seizure-free throughout the follow-up periods.

Preoperative assessment mainly aims to identify the area that causes eclampsia, determine the extent of lesion that can be resected or disconnected, determine the surgical strategy for patients, and avoid surgical complications, especially damage to cortical function. The second-stage implantation serves as an effective means for identifying epileptic lesions, particularly in cases where imaging results are negative or show suspicious lesions with multifocal characteristics. This approach is especially valuable when radiographic results do not align with seizure symptoms and other noninvasive examinations.

In children with epilepsy, if epileptic seizures and abnormal discharges persist for an extended period, the secondary epileptic focus may be transformed into an independent epileptic focus, eventually leading to the development of generalized or multifocal epilepsy. In this study, the duration of disease course was identified as an important factor affecting postoperative efficacy (*P* = 0.047). Twelve of 13 patients (92.3%) who underwent surgery within 1 year had favorable outcomes. In a previous report, this rate reached 87.9% if the interval from the first seizure to the surgery was < 36 months and dropped to 64.7% if the interval was > 50 months [11]. Epilepsy surgery is increasingly considered for infants and younger children, with outcomes comparable to those in older children, aiming to improve the eventual development outcome. Persistent and severe seizures can cause irreversible neurological damage in children. Considering the high neural plasticity and the high potential for recovery of the brain in early life, performing epilepsy surgery in infants and young children can yield excellent outcomes [12, 13]. Asano et al. found a correlation between enhanced intelligence and the age of surgery. Specifically, a younger age of surgery, which indicates a shorter disease duration, is associated with more notable improvements in intelligence irrespective of the postoperative seizure control [14].

All children with IESS and intractable epilepsy in this study underwent imaging evaluations including MRI and PET/MRI. When the cortical abnormalities detected on MRI are well correlated with the interictal epileptic discharges recorded on video EEG, a good post-surgical outcome may be expected [15]. Patients with positive MRI findings tend to have a higher seizure-freedom rate than those with negative MRI findings [16]. Conversely, in cases where MRI findings do not reveal structural lesions, functional neuroimaging techniques such as PET can serve as a valuable tool for the preoperative assessment of IESS. This approach can substantially decrease postoperative functional deficits in children with negative MRI findings [17]. In recent years, children with negative MRI findings for epileptic foci were further assessed by single-photon emission computed tomography, PET, and EcoG. The success rate of surgery in these children is comparable to that in patients with positive MRI findings [15]. Among the 30 patients included in our study, all received MRI scanning, and abnormalities were detected in 83.3% (25/30) of the cases. MRI-positive findings refer not only to positive reports from radiologists, but also to suspected abnormalities found by our MDT team via joint discussion, specifically by surgeons via careful reading of the images as well as image postprocessing including localized abnormalities of sulcal gyrus alignment, unclear demarcation of gray and white matter, and increased cortical thickness. Subsequently, PET/MRI examination was performed in children with MRI-negative findings, revealing focal abnormalities in 96.7% of them. Moreover, 73.3% of the patients experienced a high level of seizure control following PET/MRI-guided lesion resection or disconnection. Our findings demonstrated that positive PET/MRI findings are an independent risk factor for postoperative efficacy (*P* = 0.0119). Consistent with a previous study, PET/MRI analysis can enhance the detection of small epileptogenic lesions, especially of the FCD type I, thus improving post-surgical seizure outcomes [18]. Additionally, the absence of preoperative neuroimaging and functional imaging findings does not necessarily indicate the absence of a resectable epileptic focus in children with IESS; rather, PET-MRI scanning should be considered. Even in cases where PET/MRI fails to detect epileptic lesions, some additional localization information and potential etiological insights can also be provided by this approach [19].

We also found that etiology plays a significant role in determining postoperative efficacy (*P* = 0.020). A detailed review of patients who underwent three or more resections revealed that patients with the most refractory epilepsy have no significant pathological abnormalities, suggesting that independent histopathology and genetic etiology are associated with increased recurrence risk of seizures [20].Malformation of cortical development (MCD) is the most common structural cause reported in cohort studies involving children. Most children with MCD have refractory epilepsy and developmental delay, for which surgical resection or disconnection of the lesion is the most important treatment. Surgical resection, which accounts for 81% of all surgery types, is typically prioritized as it can support localization of the EZ. Furthermore, the seizure freedom rate at 2 years postoperatively exceeds 70% [21]. In our study, three children underwent lobar or multilobar cortical resection, and the rate of effective seizure control was 100% (3/3) after 2 years of follow-up. For patients with IESS undergoing hemispherotomy, the rate of effective seizure control was 76.9% (10/13) after 2 years of follow-up, suggesting that hemispherotomy is an effective treatment for patients with drug-refractory IESS in terms of seizure outcome and prognosis (*P* < 0.046). A study examining the seizure outcome in 83 children with refractory epilepsy after hemispherectomy revealed an 83.1% (69/83) seizure-freedom rate during the 3-year follow-up period, while 16.9% (14/83) experienced epilepsy recurrence. Notably, bilateral PET abnormalities are independently associated with adverse outcomes of epileptic seizures [22]. A second hemispherotomy following failure of the first hemispherotomy is a valid option with a low incidence of postoperative complications. The reason for the failure is usually attributed to the residual fibrous connection between the two cerebral hemispheres, which requires an accurate preoperative workup. Diffusion tensor imaging can be used to identify the remaining interhemispheric connections after surgery [23]. When the EZ cannot be localized or evidence suggests multifocal lesions or generalized seizures, palliative surgery can be considered. However, palliative surgery did not control seizures in the four children with IESS in this study.

Children with MCD are at a high risk of seizure recurrence. Eleven children exhibited poor seizure control after the first surgery, which could be attributed to the surgical approach, the occult epileptogenic zone, or the underlying etiology. Eight patients subsequently underwent a second operation to achieve better seizure control, resulting in favorable outcomes with a 72.7% effective rate. The results suggest favorable outcomes in infants and young children with intractable epilepsy following reoperation and that the scope of surgical resection or disconnection is the key for a good prognosis, in consistency with previous studies [24–26]. Therefore, reoperation is recommended for children with seizures after the first surgery, especially those with MCD, to achieve more favorable outcomes.

FCD is a unique MCD subtype and a common cause of focal epilepsy in approximately 25% of children. Surgical resection can improve outcomes in 50–75% of children with FCD [27]. A study on the treatment of 51 children with drug-refractory IESS and FCD found that the seizure-freedom rate was 33.3% (7/21) in patients treated with a ketogenic diet and 73.3% (22/30) in those who underwent surgery [28]. The effective rate of seizure control in terms of postoperative histopathological type was 100% for encephalomalacia foci or scars (7/7) and 76.5% for MCD (15/19), respectively. Previous studies have reported that detection of FCD on MRI and complete resection are significant predictors for seizure control among patients with isolated FCD [29]. The correlation between the pathological type of FCD and surgical prognosis remains controversial. In this study, the type of FCD was not associated with postoperative efficacy (*P* > 0.05). The postoperative outcome of FCD is affected by many factors, including histological type, surgical localization, lesion extent, complete resection, and age [30]. Pathology is an important factor affecting the outcome of seizures; however, it has a limited effect on cognitive outcomes [31]. Patients with focal cortical lesions, especially low-grade tumors, are more likely to achieve remission than those with non-lesional focal epilepsy, multifocal lesions, or cerebral cortical dysplasia [11]. We concluded that the lesion extent is an independent risk factor affecting postoperative outcomes (*P* = 0.0003). In our study, compared to cases with extensive involvement of functional brain areas or developmental delay, cases with localized brain damage and normal development before surgery showed better long-term developmental outcomes. Surgery is an excellent option for the treatment of IESS with unilateral, focal congenital, or early-acquired cortical lesions [3].

The limitations of this study included the single-center design, retrospective descriptive analysis, small sample size, and a relatively short follow-up period. Large-scale multicenter prospective studies are warranted to provide deeper insights into the association between preoperative variables and prognostic outcomes.

Postoperative complications such as hemiparesis, intracranial infection, and intracranial hematoma may also occur after surgery. The primary purpose of IESS treatment is to control seizures, and the ultimate purpose is to improve intellectual and motor development. The cognition and motor performance were improved after surgery in 46.7% (14/30) of the children, and 40% (12/30) of them remained within the presurgical development level. Our study confirmed that epilepsy surgery does not harm motor performance in children with or without spasticity, which is consistent with previous reports [32, 33]. The improvement in intellectual and motor function may be related to the short seizure duration, favorable seizure outcomes, and reduced use of ASMs.

## Conclusions

In summary, surgery is an important treatment option for patients with drug-refractory IESS caused by structural etiology including congenital or early-acquired cortical lesions. Early preoperative evaluation and detection of EZ are essential, and a detailed etiologic analysis should be conducted for all patients with IESS or drug-refractory epilepsy. Well-grounded planning of the scope of resection or disconnection is required to prevent the recurrence of epilepsy and to maximize the potential for brain plasticity. To ensure the safety of surgery, an appropriate approach should be selected to terminate seizures and improve the prognosis of children with IESS.

## Supplementary Information

Supplementary material 1.

## Data Availability

The data that support the findings of this study are available from the corresponding author upon reasonable request.

## Abbreviations

ASM: Anti-seizure medication
ECoG: Electrocorticogram
EEG: Electroencephalography
ES: Epileptic spasms
EZ: Epileptogenic zone
FCD: Focal cortical dysplasia
IESS: Infantile epileptic spasms syndrome
ILAE: International League against Epilepsy
MCD: Malformation of cortical development
MRI: Magnetic resonance imaging
PET: Positron emission tomography
ROC: Receiver operating characteristic
