# Supplementary material for: Surgical outcomes of drug-refractory infantile epileptic spasms syndrome and related prognostic factors: a retrospective study
Source: Acta Epileptol. 2024 Nov 1;6:36. doi: 10.1186/s42494-024-00176-1 (PMC11960351; doi:10.1186/s42494-024-00176-1)

**SUPPLEMENTARY FIGURES**

**Supplementary Figure 1**. Preoperative evaluation flow chart


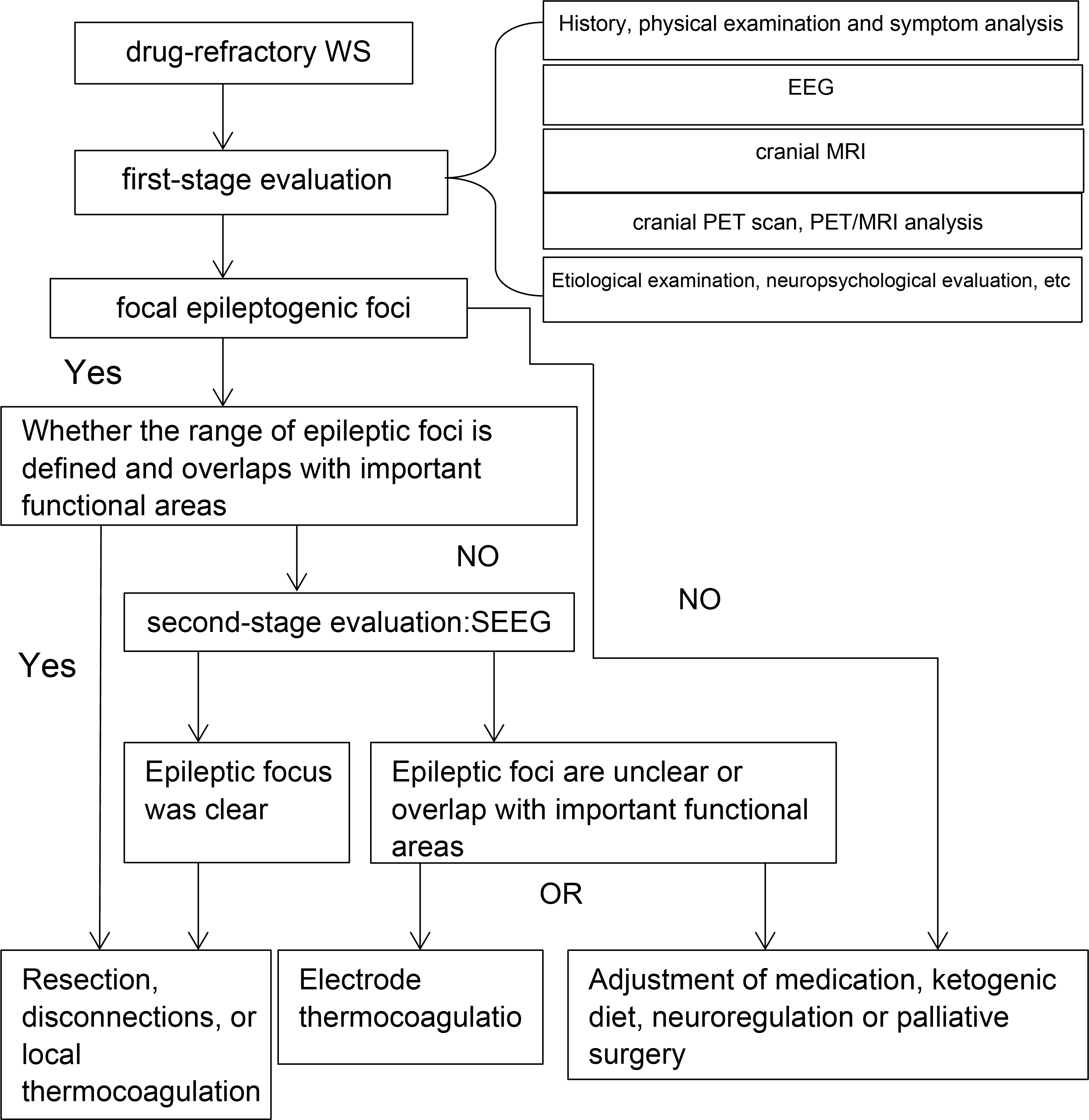


**Supplementary Figure 2**. ROC Curve


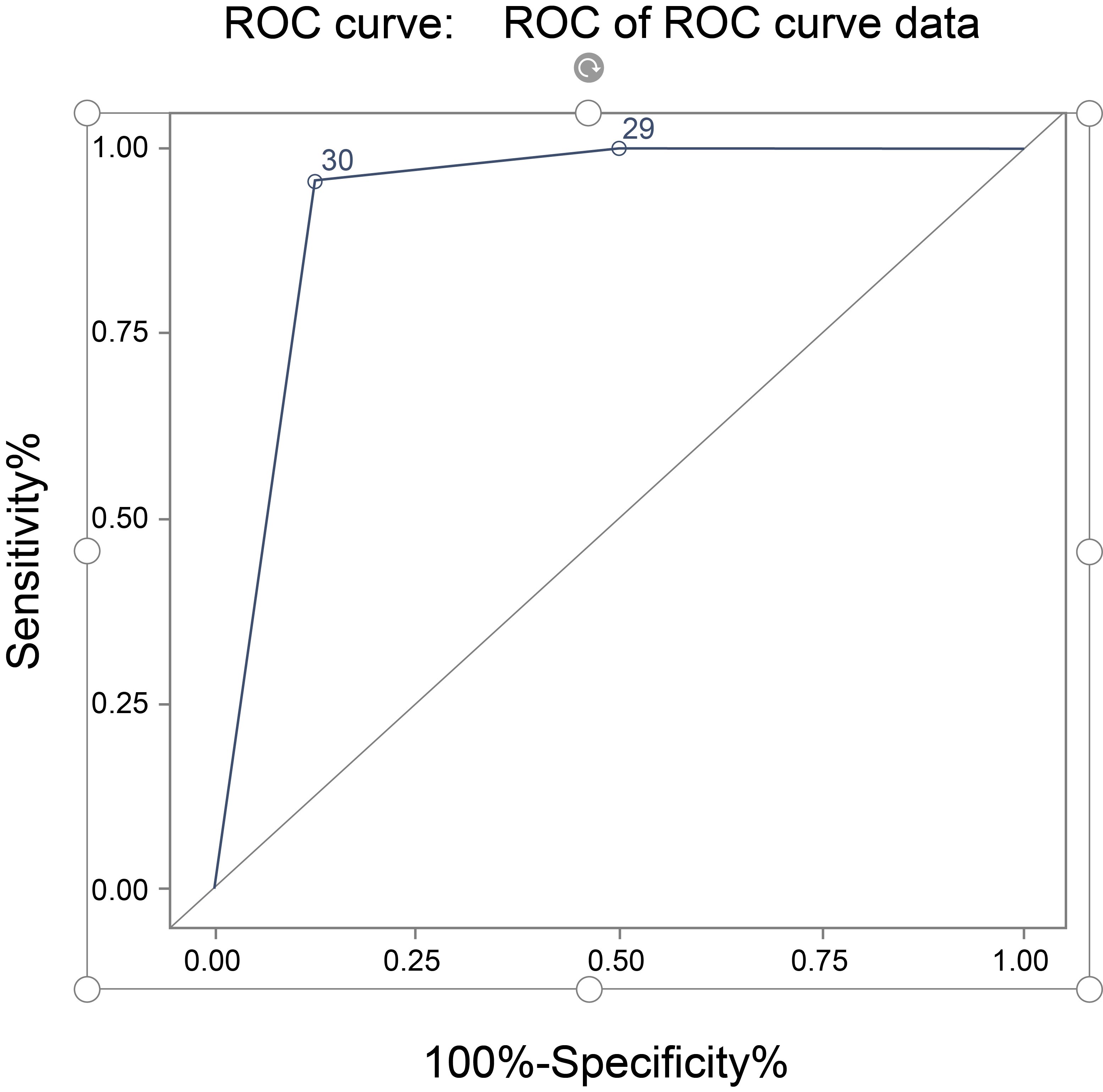

Supplement: Supplementary file 1 — Supplementary material 1. [file 42494_2024_176_MOESM1_ESM.docx]
